# The immediate ex vivo covering and filling characteristics of antibiotic-loaded resorbable calcium sulfate paste around intramedullary nails

**DOI:** 10.5194/jbji-9-261-2024

**Published:** 2024-11-07

**Authors:** Amber A. Hamilton, Jidapa Wongcharoenwatana, Jason S. Hoellwarth, Austin T. Fragomen, S. Robert Rozbruch, Taylor J. Reif

**Affiliations:** 1 Limb Lengthening and Complex Reconstruction Service, Hospital for Special Surgery, New York, NY 10021, USA; 2 Department of Orthopedic Surgery, Weill Cornell Medical College, New York, NY 10021, USA

## Abstract

**Background**: Antibiotic-laden polymethyl methacrylate (PMMA)-coated intramedullary nails (IMNs) are an effective treatment for osteomyelitis, but they pose multiple disadvantages. Antibiotic-loaded resorbable calcium sulfate (ARCS) paste is an alternative option to deliver a local antibiotic depot around IMNs, but such use has been minimally investigated. This study aimed to define the immediate covering and filling characteristics of ARCS around IMNs by using anatomic bone models. **Method**: Five tibia models (foam with cortical shell) were prepared by reaming a uniform 13 mm cylindrical path. Three 40 cc kits of ARCS (STIMULAN, Biocomposites Ltd, Keele, UK) were mixed with 3 g vancomycin and 1.2 g tobramycin powder and injected into the intramedullary canal while wet, completely filling the canal. A 10 mm × 345 mm tibial IMN was immediately inserted without interlocking screws and allowed to completely cure for 2 h. The models were then longitudinally cut without disrupting the dry ARCS covering on the nail. The ARCS was removed from the nail at five equidistant locations along the nail. The thickness of the ARCS was measured with a caliper. A repeated-measures ANOVA test was used to compare the mean width of each segment for each model. **Results**: In all five trials, the tibial canal volume surrounding the nail remained completely filled. The ARCS paste was confluent along the length of the IMN. There were no gaps or air pockets between the paste and reamed model bone. There was no statistically significant difference among the five samples at each location ( p=0.913 ) or among the five locations along the bone ( p=0.210 ). **Conclusion**: In a model setting, ARCS fully fills the intramedullary canal of a tibia and covers an IMN uniformly. Study of the in vivo material properties of ARCS may further elucidate the bone penetration as well as the clinical utility of this antibiotic depot technique.

## 1 Introduction

Infection of a bone and adjacent soft tissue presents multiple challenges including how to definitively diagnose the infection, whether and to what extent surgical debridement is required, how to support structurally compromised bone, and how to manage retained or new metal implants to prevent ongoing infection (Townsend and Koç, 2023). Most orthopedic implants offer no intrinsic ability to fight infection (Makridis et al., 2013); therefore, early removal of colonized implants is generally recommended (Haseeb et al., 2017). However, this is challenging should the bone still require stabilization. Familiar options to maintain stability include using an external fixator (Lam et al., 2019) or intramedullary nail (IMN) coated with polymethyl methacrylate (PMMA) (Makhdom et al., 2020). External fixators can lead to new pin infections, increased patient accommodation, and require eventual removal, compared with internal intramedullary fixation (Modin et al., 2009). PMMA IMN techniques present technical challenges and can require a narrower nail diameter (Hake et al., 2015). An alternative antibiotic depot option is antibiotic-loaded resorbable calcium sulfate (ARCS) paste around an implant (Downey et al., 2022). When inserted into an intramedullary canal in the liquid phase followed by IMN insertion (Rivera et al., 2021), ARCS provides a gradually absorbed (Oh et al., 1998) and eluting antibiotic depot (Mereddy et al., 2023) and is compatible with static or telescopic IMNs (Downey et al., 2022). Presumably, the technique also reduces or eliminates intraosseous dead space, at least temporarily, by conforming to the intramedullary canal. However, the assumption of the uniformity and consistency of ARCS around the IMN has never been investigated.

This work was inspired by the publication of Rivera et al. (2021). The aforementioned work had two key points: first, it described the surgical technique of preparing an ARCS paste (STIMULAN, Biocomposites Ltd, Keele, UK) laden with vancomycin, tobramycin, and/or daptomycin that was injected into an intramedullary canal prior to the insertion of a motorized intramedullary nail; second, the article confirmed that the nail's telescopic function (lengthening or compression) remained uninhibited in vivo. A related article by Downey et al. (2022) further supported the technique by demonstrating infection eradication equivalent to the traditional PMMA IMN technique. In clinical experience, direct visual and radiographic observation of the ARCS during these surgeries has led the authors of the current article to believe that ARCS completely fills the intramedullary canal, minimizing dead space and maximizing the amount of antibiotic delivered. This is in contrast to nails covered with PMMA and allowed to cure externally: such constructs cannot fully fill the canal because that would interfere with nail passage, and some of the antibiotics are never delivered given the elution limitations of PMMA (Anagnostakos and Meyer, 2017). However, the impression that ARCS uniformly fills the canal around the IMN has never been confirmed.

Accordingly, the intent of this investigation was to describe the physical morphology of how the ARCS paste fills the intramedullary canal in the setting of a simultaneous placement of an IMN.

## 2 Methods

### 2.1 Investigation technique

The specific ARCS product used in this investigation has Conformité Européenne (CE) approval for use as a bone void filler with or without antibiotics; the United States Food and Drug Administration (FDA) has only approved it as a bone void filler.

Recognizing that, in vivo, calcium sulfate is resorbed by various body processes over time (Thomas et al., 2005) at varying rates depending on factors such as surface area and additives (Thomas and Puleo, 2009), this investigation specifically intended to define the morphology immediately upon nail insertion. Once ARCS sets into a chalk-like consistency, it holds its shape unless mechanically disrupted (it does not change shape or deteriorate appreciably under ambient conditions). Therefore, it was considered reasonable to use anatomic bone models rather than actual bones to evaluate this study's question. Foam with cortical shell tibia models (SAWBONES, Pacific Research Company, Vashon, WA, USA) that were 405 mm long and had preexisting 10 mm canal diameters were used. The intramedullary canals were further prepared by placing a guide wire centrally and then reaming with flexible non-cutting reamers in 0.5 mm increments from 10 to 13 mm. The reaming was performed to the level of the distal epiphysis. Unlike human bone, the models' metaphyseal flares were also occupied by foam filler that created a consistent canal of uniform diameter to start and also when reamed. The five tibias were prepared in succession prior to the insertion of ARCS.

**Figure 1:**
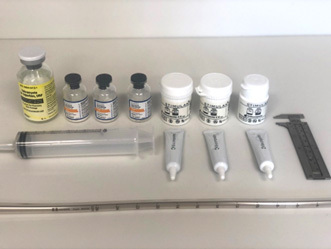
Preparation for mixing antibiotic-loaded calcium sulfate paste consisting of three STIMULAN kits, 1.2 g tobramycin, 3 g vancomycin, a 28 French chest tube, and a Toomey syringe. The caliper on the right was used for eventual measurement of the ARCS shell.

Following reaming, the canal was filled with ARCS paste. The ARCS paste was made in accordance with the manufacturer's recommendations, with modifications based on the aforementioned protocols (Rivera et al., 2021; Downey et al., 2022) and as detailed subsequently. The materials used for the ARCS preparation are shown in Fig. 1. Three ARCS powder packets (40 cc each, total 120 cc), one vial of powdered tobramycin (1.2 g), and three vials of powdered vancomycin (1 g each, total 3 g) were thoroughly mixed in a bowl. In a rapid fashion, three vials of the liquid ARCS activator along with two to three drops of saline were added to the powder. This mixture was then rapidly stirred with a plastic cement mixing spatula for 30 s to achieve a complete mix, with the liquid now injectable but maintaining a “stiff peaks” consistency. In this state, the antibiotic ARCS was quickly poured into a 60 cc Toomey syringe. A 28 French chest tube was attached to the nozzle of the syringe and air inclusion was minimized by pressurizing the ARCS within the syringe. The liquid ARCS was injected into a single tibia model's intramedullary canal by gradually pulling the chest tube up and out of the canal as filling occurred, as described in the reference articles (Rivera et al., 2021; Downey et al., 2022). This was all done under direct vision, not radiographically.

**Figure 2:**
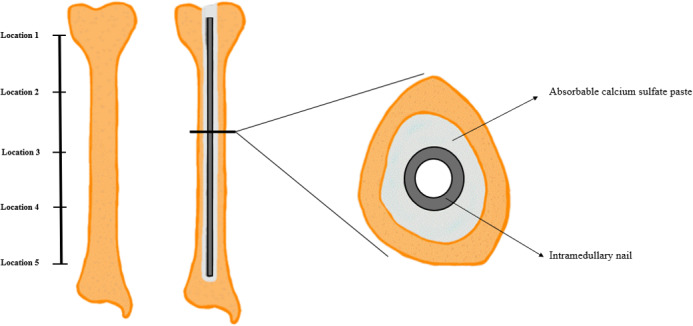
Illustration demonstrating the position of the IMN relative to the model bone after injection with absorbable calcium sulfate paste and nail insertion.

With the ARCS now in a semi-toothpaste consistency, a static tibia IMN 10 mm × 345 mm (T2 Tibia, Stryker, Kalamazoo, MI, USA) was inserted into the tibial canal using the standard nail inserter as a handle. The nail was inserted until the top of the nail had just passed the proximal cortical shell. The manufacturer recommends 8 min for the ARCS to become solid, but the addition of antibiotics hastens this to approximately 5 min. To prevent nail migration during curing, the handle was held for 10 min and then gently released. The entire construct was gently laid flat for at least another 2 h to ensure complete curing. This produced a construct as shown in Fig. 2. The entire ARCS preparation, injection, and nail insertion was performed fresh for each tibia.

Specific deviations from the reference articles (Rivera et al., 2021; Downey et al., 2022) are as follows: model tibias were used rather than real surgical procedures in human tibias; no cement gun was used, only the Toomey syringe with chest tube; no radiology was used to visualize the canal filling, visual inspection was felt to be sufficient; no cross-locking of nails with screws was performed; and static rather than motorized nails were used.

### 2.2 Analysis of samples

**Figure 3:**
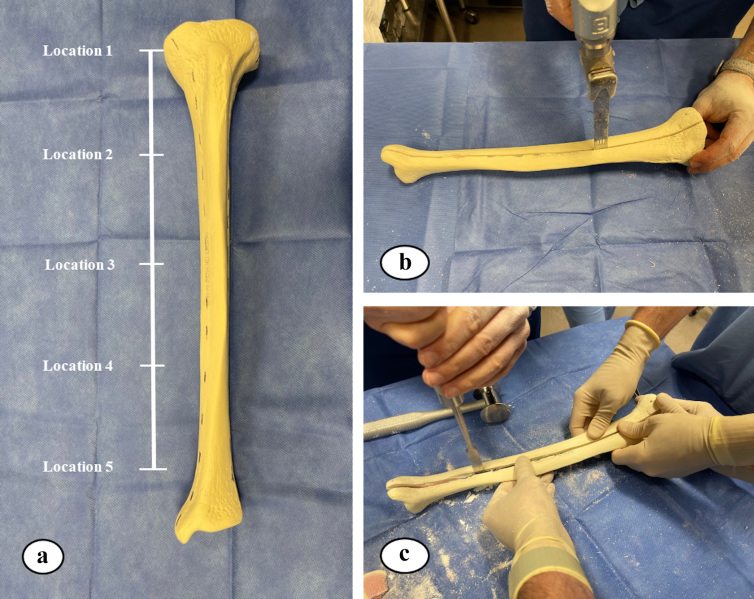
**(a)** Five equidistant locations, from proximal to distal, on the nail were measured and located. **(b, c)** A saw and osteotome were used to cut and split the SAWBONES longitudinally.

**Figure 4:**
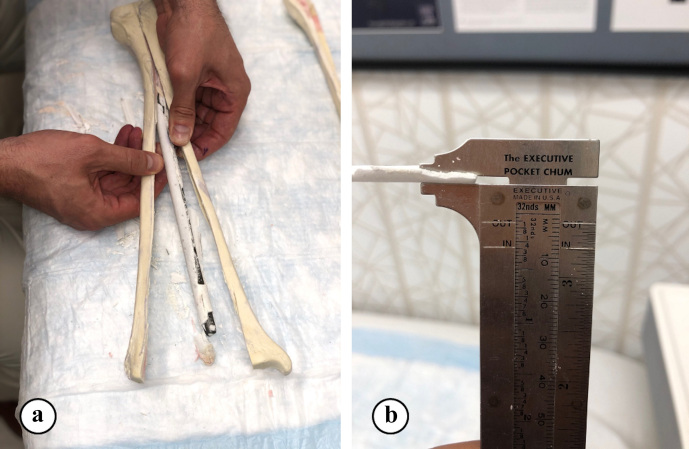
**(a)** The dry segments were collected from five locations on the nail from each of the SAWBONES. **(b)** Each segment width was measured with a fine (0.1 mm increments) caliper.

The thickness of hardened ARCS between the nail and the tibia was evaluated at five locations (Fig. 2). To extricate the ARCS–IMN construct from the bone model, the following technique was performed (Figs. 3 and 4): a powered oscillating saw (Stryker Performance Series 6125-127-90) was used to cut the SAWBONES longitudinally, minimizing disruption to the hardened ARCS. This was performed circumferentially, essentially similar to a cast removal technique, but also on the proximal and distal aspects of the tibia models. An osteotome was then used to gently wedge apart the anterior and posterior tibia model components. The anterior portion of solid ARCS was gently removed from the nail at each of the five locations. Following this, the removed ARCS thickness was measured with a caliper capable of 0.1 mm increments.

### 2.3 Statistical analysis

The mean thickness of each sample was compared as follows: each specific location was compared across all five different models, and each individual model had all five locations compared. The analysis was performed using a repeated-measures ANOVA test (DATAtab Team, 2023). Significance was set as p<0.05 .

## 3 Results

The primary aim of the study was to evaluate the immediate filling characteristics of the ARCS–IMN construct in a tibia bone model. In all five trials, the entire tibial canal volume remained completely filled, and the ARCS paste was confluent along the entire length of the IMN. There was also no shrinkage of the paste leading to gaps between the paste and the model foam, nor were there air pockets between the IMN and the paste.

**Figure 5:**
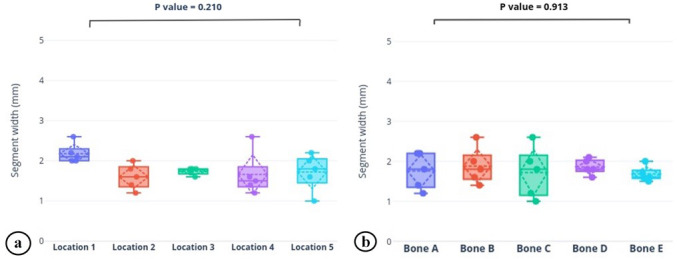
Graphical depiction of the **(a)** average dry segment width from five locations on the nail and **(b)** average dry segment width from each bone model (bone A–E). Box plots portray the median, quartiles, and range (solid line, box, and whiskers); average and standard deviation (dotted horizontal line and diamond); and each data point (solid dot) of the cohort. There were no statistically significant differences in either parameter.

Table 1 and Fig. 5 show the results of the primary aim. There were no statistically significant differences among each of the five samples ( p=0.913 ), nor were there differences among any of the five locations along the bone ( p=0.210 ). As a reminder, the model tibia's canal was a uniform cylinder after reaming because the metaphyseal flares are also uniformly filled with foam in the model.

**Table 1:** Dried paste segment width from each location (location 1–5) from five bone models (bone A–E).

	Segment width (mm)						Bone p value
	Location 1	Location 2	Location 3	Location 4	Location 5	Mean ± SD	
Bone A	2.2	1.2	1.8	1.4	2.2	1.8±0.4	0.913
Bone B	2.0	1.4	1.6	2.6	1.8	1.9±0.4	
Bone C	2.6	2.0	1.8	1.2	1.0	1.7±0.6	
Bone D	2.1	1.8	1.8	1.6	2.0	1.9±0.2	
Bone E	2.0	1.6	1.7	1.5	1.6	1.7±0.2	
Mean ± SD	2.2±0.3	1.6±0.3	1.7±0.1	1.7±0.6	1.7±0.5		
Location p value	0.210						

Data in columns are presented as the mean ± SD, and p<0.05 was considered significant.

## 4 Discussion

The immediate intramedullary filling characteristics are uniform: there were no statistical differences in the ARCS mantle thickness among the five trials at any single location ( p=0.913 ), nor were there differences in the mean thickness among the five locations ( p=0.210 ). The ARCS mantle uniformly and completely fills the intramedullary canal around the nail. These observations suggest that, when ARCS is injected into the intramedullary canal in a wet phase, followed by insertion of an IMN while the ARCS is still wet, thorough filling of the canal can be achieved. This knowledge improves the understanding of the use of ARCS as an option for providing a local antibiotic depot in situations where implants remain necessary, and this technique could be a reasonable alternative among the growing field of options for infection management in the setting of local implants. Specifically, this study contributes to the understanding of the immediate physical behavior of ARCS paste surrounding IMNs. The following discussion will contextualize this study by presenting the fundamentals of fixation for infected situations, some principles of PMMA and ARCS for such situations, and some potential implant modifications.

The treatment or prevention of local bone or soft-tissue infection in situations demanding fixation remains challenging. Trampuz and Zimmerli (2006) report that a majority of such infections acquired during trauma-related fixation are caused by staphylococci. Upwards of a 70 % union rate can occur if (1) debridement with deep cultures is performed (to direct antibiotic therapy), (2) implant stability is ensured (to confer a physically stable healing environment), and (3) local and systemic targeted antibiotics are provided (Trampuz and Zimmerli, 2006; Zimmerli, 2014; Berkes et al., 2010). Once the fracture has united, implant removal should be considered, as this reduces a risk of late reinfection (Brahme et al., 2023). Bacteria often form a biofilm on foreign material, such as metal orthopedic implants; even with antibiotic penetrance of the biofilm, the effectiveness of the antibiotics on the bacteria are often greatly reduced (such as due to dormancy) and the bacteria can remain a long-term source of potential or actual recurrent infection (McConoughey et al., 2014; Arciola et al., 2015).

External fixation, in particular Ilizarov (Barbarossa et al., 2001) or hexapod fixators (Napora et al., 2017), can reduce immediate and long-term bacterial contamination in the healing zone. If the infection appears contained to a small region, fixation can be applied remote to this in the same bone, minimizing the risk of local inoculation of the implants. As external fixation is relatively temporary by design, even if the transcutaneous implants become contaminated, they are eventually removed, at which time routes of ingress close. External fixators permit versatile stabilization and correction strategies, such as intentional deformity (Hernández-Irizarry et al., 2021), and can change position gradually which reduces local tissue stress. The major disadvantage of external fixation is the increased patient management burden (Abulaiti et al., 2017), which can be reduced by replacing the external fixator with an internal nail (Rozbruch et al., 2008) or plate (Harbacheuski et al., 2012).

A nail covered with antibiotic-laden PMMA can also provide fixation in an infected setting. PMMA is a relatively versatile antibiotic carrier (Magnan et al., 2013) and conforms to complex anatomy when inserted in soft phase or can be molded to a desired rigid shape (such as beads) prior to insertion (DeCoster and Bozorgnia, 2008) or even around temporary joint replacement implants (Haddad et al., 2000). Antibiotic elution from PMMA occurs in an exponential-decay pattern (Amin et al., 2012), generally mostly expired by 7–14 d (Anagnostakos et al., 2009), and is affected by volume and surface area and the specific antibiotic or PMMA formulation used (Duey et al., 2012). It has also proven successful for treating infection in many orthopedic situations such as long-bone fractures (Downey et al., 2022) and arthroplasty (Wang et al., 2013; Jacobs et al., 2009). However, the PMMA might eventually become another nidus for infection (Ganta et al., 2022). There are many ways to fabricate a nail with antibiotic PMMA (Kim et al., 2014; Koury et al., 2017), but a common drawback is that the nail must be a thinner diameter in order to provide space for the PMMA inside the intramedullary canal, and removal can be complicated by the PMMA debonding from the nail (Abalkhail et al., 2022). The technique used in this study – using ARCS in association with an IMN – evolved directly from experience using PMMA with IMNs.

The effectiveness of ARCS as an antibiotic delivery agent has previously been established. Gauland (2011) reported that 93.8 % of 323 osteomyelitis patients healed after using ARCS with vancomycin and gentamicin after surgical debridement. Ferguson reported that 191 of 195 (97.9 %) cases treated with debridement and ARCS eradicated infection; bone defect filling was complete in only 4.4 % of cases, emphasizing that ARCS has limited structural benefit. In a prospective trial of 30 patients treated with absorbable antibiotic-loaded calcium sulfate vs. PMMA, McKee identified an identical 86 % infection eradication rate, an almost identical ( 7/8=88 % vs. 6/8=75 %) fracture healing rate, but a different ( 7/14=50 % vs. 15/14=110 %) reoperation rate (McKee et al., 2002, 2010). Chang reported 94 % vs. 59 % healing ( p<0.05 ) when using ARCS vs. debridement alone (Chang et al., 2007). A review by Jacob et al.(2023) identified a 92.9 % bony-union rate and a 95.1 % rate of infection eradication. Compared with gentamicin-loaded PMMA beads, Raglan et al. (2015) demonstrated that ARCS paste was associated with improved healing times and a reduced length of stay. Patil et al. (2021) and Qin et al. (2019) demonstrated over 90 % infection eradication using ARCS for diabetic foot. ARCS also improves prosthetic joint infection eradication (Reinisch et al., 2022). Because of its gradual absorption, ARCS can hold high antibacterial concentrations in a space, such as a joint, for up to 3 weeks (Wiesli et al., 2022); however, different antibiotics elute differently, so dosing must be considered individually for patients (Wahl et al., 2011). Although not considered consistently achieved, biofilm could potentially be eradicated via only prolonged antibiotic exposure (Baeza et al., 2019). Although slightly beyond the scope of this study, very informative recent studies identify that bacteria can invade the peri-implant bone, specifically the osteocyte lacuna–canaliculi network (Jensen et al., 2023; de Mesy Bentley et al., 2017; Jensen et al., 2017; de Mesy Bentley et al., 2018). We speculate that antibacterial therapy may possibly be more effective if ARCS could also penetrate these spaces. This hypothesis is not examined in the current study but may deserve appropriate subsequent investigation.

There are some emerging options in the field of bone fixation in the setting of infection. Surface coatings are an appealing category of implants (Darouiche, 2007). A recent review of intramedullary nail coatings identified that the commercially available options are PMMA or gentamicin-coated nails (Walter et al., 2022). Coating nails with gold, silver, or palladium is an additional option (Karupiah et al., 2022; Alt, 2022). Reviews of silver-coated arthroplasty implants emphasize the limited evidence with respect to efficacy and the need to prevent toxicity (Diez-Escudero and Hailer, 2021; Wyatt et al., 2019). As machining techniques continue to improve, it is becoming increasingly evident that smoother implant surfaces prevent the adhesion of bacteria such as *Streptococcus sanguis* (Pereira da Silva et al., 2005) and staphylococci species (Wu et al., 2011). Nanotubules on the surface of implants can also act as specific antibiotic carriers, providing antimicrobial action without measurable additional volume (Yang et al., 2016; Wang et al., 2021). One additional category of implant surface modification to prevent infection is ion doping. Copper and zinc cation doping of cobalt alloy implants shows potential to prevent infection of *Staphylococcus aureus* and *Pseudomonas aeruginosa* (Totea et al., 2015).

There are important limitations to this study. The most apparent is that a plastic bone model was used rather than a cadaver or living bones, which is missing metaphyseal flares and any form of canaliculi. However, this was deliberately chosen in order to minimize confounding variables with the canal preparation and evaluation of ARCS. Future studies with cadavers or with living patients may be appropriate, perhaps using computational tomography or magnetic resonance imaging to evaluate the ARCS mantle around the implant and penetration into the surrounding tissue, ideally in a time-dependent manner. Another limitation is that this study only utilized a relatively straight intramedullary canal of a tibia, rather than a curved canal such as in a femur; this could alter the ARCS mantle thickness due to the obligate geometry of a curved implant in a curved bone. Cutting the model bone theoretically could have resulted in disruption of the ARCS mantle; however, in practice, the medial mantle integrity seemed to be very highly preserved. A modification to bisect the bone prior to ARCS filling and nail insertion could be considered, but this would likely lead to substantial ARCS leakage. An alternate strategy for the evaluation of thickness could have been to use computational tomography. The setting characteristics at different ARCS viscosities could have been evaluated, such as with thicker ARCS, but it was considered optimal to evaluate the most liquid consistency available to provide the greatest potential uniformity and because this is the recommended surgical technique. As a technical reminder, ARCS may provide increased early stabilization of unstable bone by interdigitation between the bone and a nail, but the construct may lose stability upon resorption; the biomechanics of this potential scenario have not been studied. The following is another important limitation: the elution of antibiotics from a calcium sulfate carrier is in vivo poorly characterized; clinicians should consider whether the paste may not harden due to local bleeding (and thereby result in spiked systemic levels) and perhaps adjust the dosage (Wahl et al., 2011).

## 5 Conclusion

In a model setting, ARCS fills the intramedullary canal of a tibia and covers an IMN uniformly. Further studies of the material properties of ARCS with IMNs in human bone may help understand the potential utility of this phenomenon in situations requiring orthopedic fixation in the setting of local infection.

## Data Availability

The data used in this study are available in Table 1.

## Code availability

Statistical analysis was carried out with https://datatab.net/ (DATAtab Team, 2023).

## References

[bib1.bib1] Abalkhail TB, Elhessy AH, Conway JD (2022). Removal of Antibiotic Cement-Coated Interlocking Nails. J Orthop Trauma.

[bib1.bib2] Abulaiti A, Yilihamu Y, Yasheng T, Alike Y, Yusufu A (2017). The psychological impact of external fixation using the Ilizarov or Orthofix LRS method to treat tibial osteomyelitis with a bone defect. Injury.

[bib1.bib3] Alt V (2022). Treatment of an infected nonunion with additional fresh fracture of the femur with a silver-coated intramedullary nail: A case report. Trauma Case Reports.

[bib1.bib4] Amin TJ, Lamping JW, Hendricks KJ, McIff TE (2012). Increasing the Elution of Vancomycin from High-Dose Antibiotic-Loaded Bone Cement: A Novel Preparation Technique. J Bone Joint Surg Am.

[bib1.bib5] Anagnostakos K, Meyer C (2017). Antibiotic Elution from Hip and Knee Acrylic Bone Cement Spacers: A Systematic Review. BioMed Res Int.

[bib1.bib6] Anagnostakos K, Wilmes P, Schmitt E, Kelm J (2009). Elution of gentamicin and vancomycin from polymethylmethacrylate beads and hip spacers in vivo. Acta Orthop.

[bib1.bib7] Arciola CR, Campoccia D, Ehrlich GD, Montanaro L (2015). Biofilm-Based Implant Infections in Orthopaedics. Adv Exp Med Biol.

[bib1.bib8] Baeza J, Cury MB, Fleischman A, Ferrando A, Fuertes M, Goswami K, Lidgren L, Linke P, Manrique J, Makar G, McLaren A, Moriarty TF, Ren Q, Vince K, Wahl P, Webb J, Winkler H, Witsø E, Young S (2019). General Assembly, Prevention, Local Antimicrobials: Proceedings of International Consensus on Orthopedic Infections. J Arthroplasty.

[bib1.bib9] Barbarossa V, Matković BR, Vučić N, Bielen M, Gluhinić M (2001). Treatment of osteomyelitis and infected non-union of the femur by a modified Ilizarov technique: Follow-up study. Croat Med J.

[bib1.bib10] Berkes M, Obremskey WT, Scannell B, Ellington JK, Hymes RA, Bosse M (2010). Maintenance of Hardware After Early Postoperative Infection Following Fracture Internal Fixation. J Bone Joint Surg Am.

[bib1.bib11] Brahme IS, Hu CH, Cole PA (2023). Infection from an Iliosacral Screw 16 Years Postoperatively in Demolition Derby Umpire Crushed Between 2 Cars: A Case Report. JBJS Case Connector.

[bib1.bib12] Chang W, Colangeli M, Colangeli S, Di Bella C, Gozzi E, Donati D (2007). Adult osteomyelitis: Debridement versus debridement plus Osteoset T^®^ pellets. Acta Orthop Belg.

[bib1.bib13] Darouiche RO (2007). Antimicrobial coating of devices for prevention of infection: Principles and protection. Int J Artif Organs.

[bib1.bib14] DATAtab team (2023). DATAtab eU Graz, Austria, EU [data set].

[bib1.bib15] DeCoster TA, Bozorgnia S (2008). Antibiotic beads. J Am Acad Orthop Sur.

[bib1.bib16] de Mesy Bentley KL, Trombetta R, Nishitani K, Bello-Irizarry SN, Ninomiya M, Zhang L, Chung HL, McGrath JL, Daiss JL, Awad HA, Kates SL, Schwarz EM (2017). Evidence of Staphylococcus Aureus Deformation, Proliferation, and Migration in Canaliculi of Live Cortical Bone in Murine Models of Osteomyelitis. J Bone Miner Res.

[bib1.bib17] de Mesy Bentley KL, MacDonald A, Schwarz EM, Oh I (2018). Chronic Osteomyelitis with Staphylococcus aureus Deformation in Submicron Canaliculi of Osteocytes A Case Report. JBJS Case Connector.

[bib1.bib18] Diez-Escudero A, Hailer NP (2021). The role of silver coating for arthroplasty components. J Bone Joint Surg Br.

[bib1.bib19] Downey E-A, Jaime KM, Reif TJ, Makhdom AM, Rozbruch SR, Fragomen AT (2022). Prophylaxis and treatment of infection in long bones using an antibiotic-loaded ceramic coating with interlocking intramedullary nails. J Bone Joint Infect.

[bib1.bib20] Duey RE, Chong ACM, McQueen DA, Womack JL, Song Z, Steinberger TA, Wooley PH (2012). Mechanical properties and elution characteristics of polymethylmethacrylate bone cement impregnated with antibiotics for various surface area and volume constructs. The Iowa Orthopaedic Journal.

[bib1.bib21] Ganta A, Merrell LA, Adams J, Konda SR, Egol KA (2022). Retention of Antibiotic Cement Delivery Implants in Orthopedic Infection Associated With United Fractures Does Not Increase Recurrence Risk. J Orthop Trauma.

[bib1.bib22] Gauland C (2011). Managing lower-extremity osteomyelitis locally with surgical debridement and synthetic calcium sulfate antibiotic tablets. Adv Skin Wound Care.

[bib1.bib23] Haddad FS, Masri BA, Campbell D, Mcgraw RW, Beauchamp CP, Duncan CP (2000). The PROSTALAC functional spacer in two-stage revision for infected knee replacements. J Bone Joint Surg Br.

[bib1.bib24] Hake ME, Young H, Hak DJ, Stahel PF, Hammerberg EM, Mauffrey C (2015). Local antibiotic therapy strategies in orthopaedic trauma: Practical tips and tricks and review of the literature. Injury.

[bib1.bib25] Harbacheuski R, Fragomen AT, Rozbruch SR (2012). Does Lengthening and Then Plating (LAP) Shorten Duration of External Fixation?. Clin Orthop Relat R.

[bib1.bib26] Haseeb M, Farooq Butt M, Altaf T, Muzaffar K, Gupta A, Jallu A (2017). Indications of Implant Removal: A Study of 83 Cases. Int J Health Sci.

[bib1.bib27] Hernández-Irizarry R, Quinnan SM, Reid JS, Toney CB, Rozbruch SR, Lezak B, Fragomen AT (2021). Intentional Temporary Limb Deformation for Closure of Soft-Tissue Defects in Open Tibial Fractures. J Orthop Trauma.

[bib1.bib28] Jacob CC, Daw JH, Santiago-Torres J (2023). The efficacy of antibiotic-impregnated calcium sulfate (AICS) in the treatment of infected non-union and fracture-related infection: a systematic review. J Bone Joint Infect.

[bib1.bib29] Jacobs C, Christensen CP, Berend ME (2009). Static and Mobile Antibiotic-impregnated Cement Spacers for the Management of Prosthetic Joint Infection. J Am Acad Orthop Sur.

[bib1.bib30] Jensen LK, Koch J, Aalbæk B, Moodley A, Bjarnsholt T, Kragh KN, Petersen A, Jensen HE (2017). Early implant-associated osteomyelitis results in a peri-implanted bacterial reservoir. APMIS, Acta Path Micro Im.

[bib1.bib31] Jensen LK, Birch JM, Jensen HE, Kirketerp-Møller K, Gottlieb H (2023). Bacterial invasion of the submicron osteocyte lacuna–canaliculi network (OLCN), a part of osteomyelitis disease biology. APMIS, Acta Path Micro Im.

[bib1.bib32] Karupiah T, Yong AP, Ong ZW, Tan HK, Tang WC, Salam HB (2022). Use of a Novel Anti-Infective Noble Metal Alloy-Coated Titanium Orthopedic Nail in Patients with Open Fractures: A Case Series from Malaysia. Antibiotics (Basel).

[bib1.bib33] Kim JW, Cuellar DO, Hao J, Seligson D, Mauffrey C (2014). Custom-made antibiotic cement nails: A comparative study of different fabrication techniques. Injury.

[bib1.bib34] Koury KL, Hwang JS, Sirkin M (2017). The Antibiotic Nail in the Treatment of Long Bone Infection: Technique and Results. Orthop Clinics North Am.

[bib1.bib35] Lam A, Richardson SS, Buksbaum J, Markowitz J, Henry MW, Miller AO, Rozbruch SR, Fragomen AT (2019). Chronic Osteomyelitis of the tibia and ankle treated with Limb Salvage Reconstruction. J Bone Joint Infect.

[bib1.bib36] Magnan B, Bondi M, Maluta T, Samaila E, Schirru L, Dall'Oca C (2013). Acrylic bone cement: current concept review. Musculoskeletal Surgery.

[bib1.bib37] Makhdom AM, Buksbaum J, Rozbruch SR, Cunha RD, Fragomen AT (2020). Antibiotic Cement-Coated interlocking Intramedullary Nails in the Treatment of Septic Complex Lower Extremity Reconstruction; A Retrospective Analysis with Two year Minimum Follow up. J Bone Joint Infect.

[bib1.bib38] Makridis KG, Tosounidis T, Giannoudis PV (2013). Management of infection after intramedullary nailing of long bone fractures: treatment protocols and outcomes. Open Orthop J.

[bib1.bib39] McConoughey SJ, Howlin R, Granger JF, Manring MM, Calhoun JH, Shirtliff M, Kathju S, Stoodley P (2014). Biofilms in periprosthetic orthopedic infections. Future Microbiol.

[bib1.bib40] McKee MD, Wild LM, Schemitsch EH, Waddell JP (2002). The Use of an Antibiotic-Impregnated, Osteoconductive, Bioabsorbable Bone Substitute in the Treatment of Infected Long Bone Defects: Early Results of a Prospective Trial. J Orthop Trauma.

[bib1.bib41] McKee MD, Li-Bland EA, Wild LM, Schemitsch EH (2010). A Prospective, Randomized Clinical Trial Comparing an Antibiotic-Impregnated Bioabsorbable Bone Substitute With Standard Antibiotic-Impregnated Cement Beads in the Treatment of Chronic Osteomyelitis and Infected Nonunion. J Orthop Trauma.

[bib1.bib42] Mereddy P, Nallamilli SR, Gowda VP, Kasha S, Godey SK, Nallamilli RR, Rohit GP, Meda VG (2023). The use of Stimulan in bone and joint infections: A prospective multicentre study. Bone Joint Open.

[bib1.bib43] Modin M, Ramos T, Stomberg MW (2009). Postoperative impact of daily life after primary treatment of proximal/distal tibiafracture with Ilizarov external fixation. J Clin Nurs.

[bib1.bib44] Napora JK, Weinberg DS, Eagle BA, Kaufman BR, Sontich JK (2017). Hexapod Frame Stacked Transport for Tibial Infected Nonunions With Bone Loss: Analysis of Use of Adjunctive Stability. J Orthop Trauma.

[bib1.bib45] Oh CW, Kim PT, Ihn JC (1998). The use of calcium sulfate as a bone substitute. J Orthop Surg (Hong Kong).

[bib1.bib46] Patil P, Singh R, Agarwal A, Wadhwa R, Bal A, Vaidya S (2021). Diabetic Foot Ulcers and Osteomyelitis: Use of Biodegradable Calcium Sulfate Beads Impregnated With Antibiotics for Treatment of Multidrug-Resistant Organisms. Wounds.

[bib1.bib47] Pereira Da Silva CHF, Vidigal GM, De Uzeda M, De Almeida Soares G (2005). Influence of titanium surface roughness on attachment of streptococcus sanguis: An in vitro study. Implant Dent.

[bib1.bib48] Qin CH, Zhou CH, Song HJ, Cheng GY, Zhang HA, Fang J, Tao R (2019). Infected bone resection plus adjuvant antibiotic-impregnated calcium sulfate versus infected bone resection alone in the treatment of diabetic forefoot osteomyelitis. BMC Musculoskelet Dis.

[bib1.bib49] Raglan M, Dhar S, Scammell B (2015). Is Stimulan (synthetic calcium sulphate tablets impregnated with antibiotics) superior in the management of diabetic foot ulcers with osteomyelitis compared with standard treatment?. Orthop Proceed.

[bib1.bib50] Reinisch K, Schläppi M, Meier C, Wahl P (2022). Local antibiotic treatment with calcium sulfate as carrier material improves the outcome of debridement, antibiotics, and implant retention procedures for periprosthetic joint infections after hip arthroplasty – a retrospective study. J Bone Joint Infect.

[bib1.bib51] Rivera JC, McClure PK, Fragomen AT, Mehta S, Rozbruch SR, Conway JD (2021). Intramedullary antibiotic depot does not preclude successful intramedullary lengthening or compression. J. Orthop. Trauma.

[bib1.bib52] Rozbruch SR, Kleinman D, Fragomen AT, Ilizarov S (2008). Limb Lengthening and Then Insertion of an Intramedullary Nail: A Case-matched Comparison. Clin Orthop Related R.

[bib1.bib53] Thomas MV, Puleo DA (2009). Calcium sulfate: Properties and clinical applications. J Biomed Mater Res B.

[bib1.bib54] Thomas MV, Puleo DA, Al-Sabbagh M (2005). Calcium sulfate: A review. J Long-Term Eff Med.

[bib1.bib55] Totea G, Ionita D, Demetrescu I (2015). Influence of Doping Ions on the Antibacterial Activity of Biomimetic Coating on CoCrMo Alloy. J Bionic Eng.

[bib1.bib56] Townsend O, Koç T (2023). Diagnosis and management of lower limb osteomyelitis. Surgery (Oxford).

[bib1.bib57] Trampuz A, Zimmerli W (2006). Diagnosis and Treatment of Infections Associated with Fracture-Fixation Devices. Injury.

[bib1.bib58] Wahl P, Livio F, Jacobi M, Gautier E, Buclin T (2011). Systemic exposure to tobramycin after local antibiotic treatment with calcium sulphate as carrier material. Arch Orthop Traum Su.

[bib1.bib59] Walter N, Rupp M, Krückel J, Alt V (2022). Individual and commercially available antimicrobial coatings for intramedullary nails for the treatment of infected long bone non-unions – a systematic review. Injury.

[bib1.bib60] Wang J, Zhu C, Cheng T, Peng X, Zhang W, Qin H, Zhang X (2013). A systematic review and meta-analysis of antibiotic-impregnated bone cement use in primary total hip or knee arthroplasty. PloS One.

[bib1.bib61] Wang K, Jin H, Song Q, Huo J, Zhang J, Li P (2021). Titanium dioxide nanotubes as drug carriers for infection control and osteogenesis of bone implants. Drug Deliv Transl Re.

[bib1.bib62] Wiesli MG, Livio F, Achermann Y, Gautier E, Wahl P (2022). Wound fluid ceftriaxone concentrations after local application with calcium sulphate as carrier material in the treatment of orthopaedic device-associated hip infections. Bone Joint Res.

[bib1.bib63] Wu Y, Zitelli JP, TenHuisen KS, Yu X, Libera MR (2011). Differential response of Staphylococci and osteoblasts to varying titanium surface roughness. Biomaterials.

[bib1.bib64] Wyatt MC, Foxall-Smith M, Roberton A, Beswick A, Kieser DC, Whitehouse MR (2019). The use of silver coating in hip megaprostheses: a systematic review. HIP Int.

[bib1.bib65] Yang Y, Ao HY, Yang SB, Wang YG, Lin WT, Yu ZF, Tang TT (2016). In vivo evaluation of the anti-infection potential of gentamicin-loaded nanotubes on titania implants. Int J Nanomed.

[bib1.bib66] Zimmerli W (2014). Clinical presentation and treatment of orthopaedic implant-associated infection. J Intern Med.

